# The Differentiation of Human Adipose-Derived Stem Cells towards a Urothelium-Like Phenotype *In Vitro* and the Dynamic Temporal Changes of Related Cytokines by Both Paracrine and Autocrine Signal Regulation

**DOI:** 10.1371/journal.pone.0095583

**Published:** 2014-04-21

**Authors:** Ming Zhang, Ming-xi Xu, Zhe Zhou, Ke Zhang, Juan Zhou, Yang Zhao, Zhong Wang, Mu-jun Lu

**Affiliations:** Department of Urology, Ninth People’s Hospital, School of Medicine, Shanghai Jiaotong University, Shanghai, China; University of California, Merced, United States of America

## Abstract

**Purpose:**

To investigate the differentiation ability of human adipose-derived stem cells (ASCs) towards urothelium-like cells in vitro and the dynamic changes of related cytokines and cytokine receptors in the culture medium.

**Materials and Methods:**

The ASCs were induced using both conditioned media (CM) and the transwell co-culture system with an immortalized urothelium cell line (SV-HUC-1,HUC) for 21 days. Protein and mRNA expression of the mature urothelium specific markers uroplakin-IA (UP-1A) and uroplakin-II (UP-II) were detected by immunofluorescence and quantitative real-time PCR, respectively. Array detection was used to screen 41 cytokines and receptors in the upper medium of urothelium, non-induced ASCs and urothelium-induced ASCs at three time points, early (12 hours), intermediate (7 days) and late (21 days).

**Results:**

After induction for 7 days, the ASCs grown in both CM and transwell co-culture system expressed uroplakin-IA (13.54±2.00%; 17.28±1.84%) and uroplakin-II (19.49±1.73%; 13.98±1.47%). After induction for 21 days, ASCs grown in co-culture had significantly increased expression of uroplakin-IA (48.03±1.25%; 49.57±2.85%) and uroplakin-II (45.38±2.50%; 46.58±1.95%). In the upper medium of urothelium, 28 cytokines and 8 cytokine receptors had significantly higher expression than the counterpart of non-induced ASCs. After 7 days induction, the expression of 22 cytokines and 8 cytokine receptors was significantly elevated in the upper medium of induced ASCs compared to non-induced ASCs. At the early and intermediate time points, ASCs secreted high levels of relative cytokines and soluble receptors, but their expressions decreased significantly at the late time point.

**Conclusion:**

The adipose-derived stem cells have the potential to be differentiated into urothelium-like cells in vitro by both CM and transwell co-culture system with mature urothelium. Numerous cytokines and receptors were involved in the differentiation process with dynamic temporal changes by both paracrine and autocrine signal regulation. Further studies should be carried out to determine the detailed mechanism of cytokines and receptors and to enhance the urothelium differentiation efficiency of ASCs.

## Introduction

Many diseases could cause urinary tract defect, such as bladder exstrophy, malignancy, or trauma. Reconstruction of urinary tract tissue and functions by cell based tissue engineering techniques is promising. But the sources of suitable seeding cells are limited. Adipose-derived stem cells (ASCs) are multi-potent, which has led to their increased use for urological regeneration, including bladder repair [1]–[3]. Previous studies have mainly focused on the smooth muscle differentiation potential of ASCs [4]–[6]. Recently, ASCs were also studied for their urothelium-oriented inducing ability [7]–[9], but the optimal inducing conditions in vitro and the related mechanism of differentiation are still unknown.

Soluble cytokines secreted by target cells are critical induce factors for ASCs differentiation. They can modulate the fate of adjacent cells via paracrine signaling which is a key mechanism for the directed differentiation of adult stem cell towards tissue specific cells[10]. In addition to paracrine signaling, autocrine signaling is also critical in stem cell differentiation, especially for maintaining specific cell phenotype. For example, in hematopoietic differentiation, the embryonic stem cells secreted VEGF, stem cell factor (SCF) and anti-apoptotic factors to stimulate the formation of colony-forming cells[11].

In this study, we investigated the differentiation ability of ASCs toward urothelium-like cells in vitro using both conditioned medium (CM) and the transwell indirect co-culture system. Additionally, the dynamic changes of cytokine and cytokine receptor levels in the culture medium at early, intermediate and late time points during induction were detected to explore the underlying mechanism of differentiation.

## Materials and Methods

### Cell Culture

All of the experimental protocols involving human tissue and cells were approved by the Ethics Committee of Ninth People’s Hospital, School of Medicine, Shanghai Jiao Tong University. Human adipose tissues were obtained from 3 individuals aged 28, 32 and 35 undergoing liposuction procedures during plastic and reconstructive surgery at the Ninth People’s Hospital, School of Medicine, Shanghai Jiao Tong University, after obtaining each patient’s written consent. The ASCs were cultured as reported previously [7], [12], [13]. At passage number 3, the ASCs were used for the induction experiments. We used an immortalized cell line from human urinary bladder urothelium (SV-HUC-1, HUC) to induce the ASCs to differentiate towards a urothelial phenotype. HUCs were obtained from the Typical Cell Culture Collection Committee of the Chinese Academy of Sciences Library. F12K supplemented with serum at a concentration of 10% was used to culture HUC cells.

### ASCs identification

Flow cytometry analysis was used to confirm the expression of mesenchymal stem cell markers, including CD29, CD44 and CD105, and hematopoietic stem cell markers, including CD34 and CD45. Cells were analyzed on a fluorescence-activated cell sorter (FACS Calibur, BD, Franklin Lakes, NJ). Data acquisition and analysis were performed by using Cell Quest software (Becton Dickinson, Franklin Lakes, NJ). ASCs were stained with a phycoerythrin-conjugated nonspecific IgG to assess background fluorescence.

We induced ASCs at passage number 3 to osteoblasts under osteogenic inductive conditions for 3 weeks and adipose cells under adipogenic inductive conditions for 2 weeks according to previous methods [1], [7]. The cells were stained with alizarin red and Oil Red O to demonstrate the differentiation into osteoblasts and adipocytes, respectively.

### Differentiation of Human ASCs In vitro

For differentiation, ASCs were cultured with HUC-derived CM or indirectly co-cultured with HUCs using a transwell system for 1–3 weeks. The HUC-derived CM was obtained by collecting the media from cultured HUCs at 70–90% confluence every 24 hours. Before use for the ASC differentiation, the collected media were centrifuged to remove cells, filtered and diluted with an equal volume of F12K supplemented with serum at a final concentration of 2%. ASCs were cultured using CM. The medium was changed every 24 hours. We used a transwell indirect co-culture system for ASC induction with HUC according to the literature [14]. In the transwell system, there was a cell culture insert (Corning-Costar) that had a porous polyethylene terephthalate membrane with a pore size of 0.4 µm. The membrane allowed free exchange of medium while preventing cell migration between the two wells. ASCs were seeded in the bottom chamber at a density of 2×10^4^ cells/well, while HUCs were seeded on the upper side insert at the same density. Cells were cultured in medium containing 50% DMEM and 50% F12K supplemented with 2% FBS. The medium was changed every 2 days. After culturing with HUC-derived CM or indirectly co-culturing with HUCs for 7–21 days, the ASCs were collected for immunofluorescence and real-time reverse transcription polymerase chain reaction (RT-PCR) to assess their ability to differentiate toward a urothelial phenotype. Human dermal fibroblasts from normal foreskin at passage number 3 were induced with HUC-derived CM and indirectly co-culturing with HUCs for 21 days as negative controls. HUCs were cultured as positive controls.

### Immunofluorescence for ASCs Induced In vitro

The primary antibodies used were UP-IA and UP-II (goat anti-human, 1∶200, Santa Cruz, USA). A secondary antibody was conjugated with FITC (rabbit anti-goat, 1∶1000, Sigma, USA). The stained cells were examined by fluorescence microscopy, and the number of ASCs that expressed UP-IA and UP-II were determined by random sampling. We randomly selected five fields of vision to calculate the number of cells in each slide. All of the evaluations were repeated at least three times using different slides.

### RNA Isolation and Quantitative Real-time PCR

Total cellular RNA was isolated from ASCs induced by HUC-derived CM using TRIzol. Complementary DNA (cDNA) was synthesized from RNA using Reverse-Transcriptase reagents (Invitrogen) according to the manufacturer’s instructions. Quantification of mRNA for the urothelium specific marker UP-IA was performed using the Stratagene Mx3000 Real-Time PCR System and SYBR Green detection kit. A specific primer for UP-IA was used according to the literature [14]. Beta-actin, a housekeeping gene, was used as a control. Relative expression levels of UP-IA were measured by the ΔΔCT method.

### Samples Collection and Detection by Human Growth Factor Antibody Array

The soluble cytokines and cytokine receptors were detected by a human cytokine antibody array in the upper medium of urothelium, ASCs induced for 12 hours, 7 days and 21 days and non-induced ASCs. Samples were collected from three different patients. At 12 hours before collection, the urothelium and ASC cell cultures were washed twice with PBS and exposed to serum free medium. The new culture media were collected after 12 hours, centrifuged to remove cell debris and then stored at −80°C. The relative content of cytokines and cytokine receptors in the upper medium were detected by the semiquantitative Human Growth Factor Antibody Array 1(Cat# AAH-GF-G1-8) (RayBiotech, Norcross, GA), which allows detection of 41 different cytokines and cytokine receptors in one experiment according to the manufacturer’s instructions. Each sample measurements were performed in duplicate on one membrane. Three times detection were repeated for statistics analysis. Briefly, membranes were blocked with 5% BSA for 30 minutes and then incubated with culture media for 1 hour. After washed, add 1 ml working solution of biotin-conjugated anti-cytokines (provided with the kit) and incubate at room temperature for 2 hours. After washed, add 2 ml of 1,000 fold diluted HRP-conjugated streptavidin to each membrane and then incubate at room temperature for 2 hours. The signals on the membranes were detected using chemiluminescene imaging system. Each film was scanned (Scanalyze) into TIF Image, and spots were digitized into densities. The values of densities were exported into Microsoft Excel table for further calculation. All of the cytokines and cytokine receptors are listed in Table S1.

### Data Analysis

All of the data reported are expressed as the means±SD. Student’s t test was used for comparison of cytokines and cytokines receptors in the upper medium between the groups of HUC and ASCs non-induced. ANOVA and Post test was used for comparison of cytokines and cytokines receptors in the upper medium among the groups of ASCs non-induced, ASCs induced for 12 hours, ASCs induce for 7 days and ASCs induced for 21 days. The comparison of induction rate between ASCs induced for 7 days and 21 days were also used Student’s t test. A P value <0.05 was considered as statistical significant difference. Data were analyzed by SPSS 20.0.

## Results

### Identification of HUCs and ASCs

**Figure 1:**
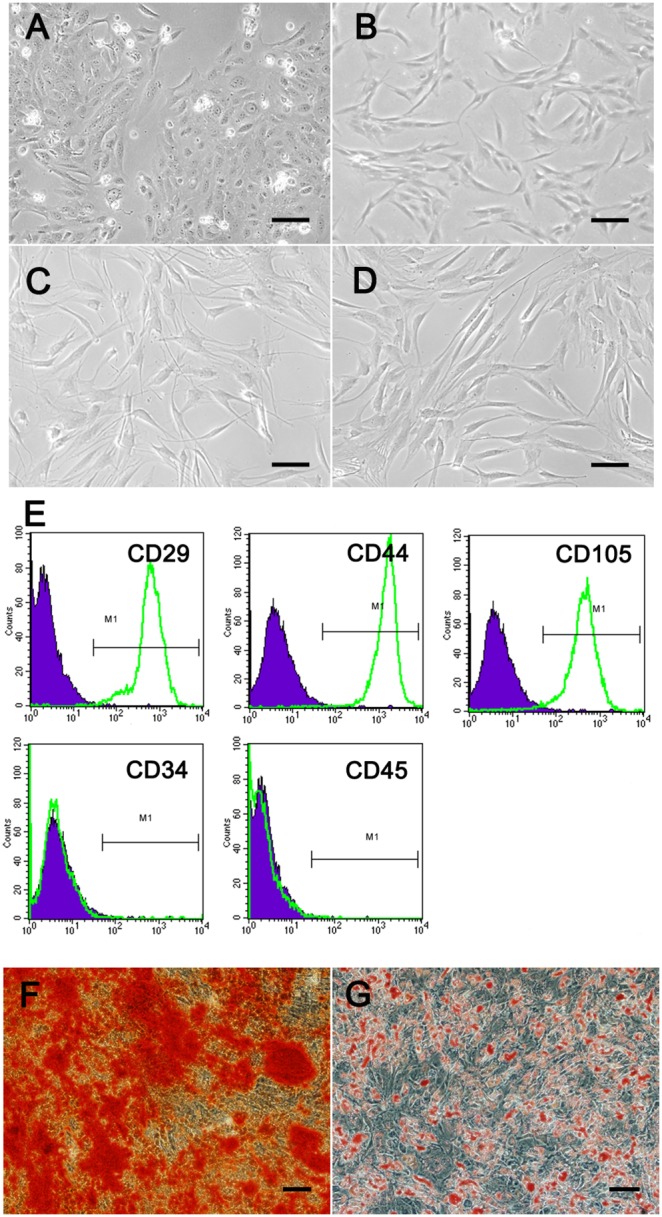
Identification of ASCs and the morphology of HUCs, ASCs and ASCs after induction. The morphology of HUCs and ASCs at passage number 3 and ASCs after induction by conditioned medium and indirect co-culture transwell system for 21 days. Identification of ASCs by flow cytometric analysis. Phase-contrast microscopy of ASCs differentiated into osteoblasts and adipocytes. A: HUCs exhibited a polygonal, cobblestone-shaped morphology. B: ASCs demonstrated spindle-shaped morphology. C: ASCs induced by conditioned medium for 21 days. D: ASCs induced by indirect co-culture transwell system for 21 days. E: ASCs from an individual donor were stained for CD29 (99.84%), CD44 (99.77%), CD105 (97.32%), CD45 (0.16%) and CD34 (0.26%). The “M1” window represents a fluorescence intensity exceeding 99% of the intensity detected in control antibody stained cells. Isotype control curves have been merged in the picture. F: Alizarin red staining of ASCs at day 21 after osteogenic induction. G: After induction with adipogenic inductive conditions for 14 days, the intracytoplasmic lipid rich droplets in ASCs stained positive for Oil Red O. Scale bar = 20 µm.

**Figure 2:**
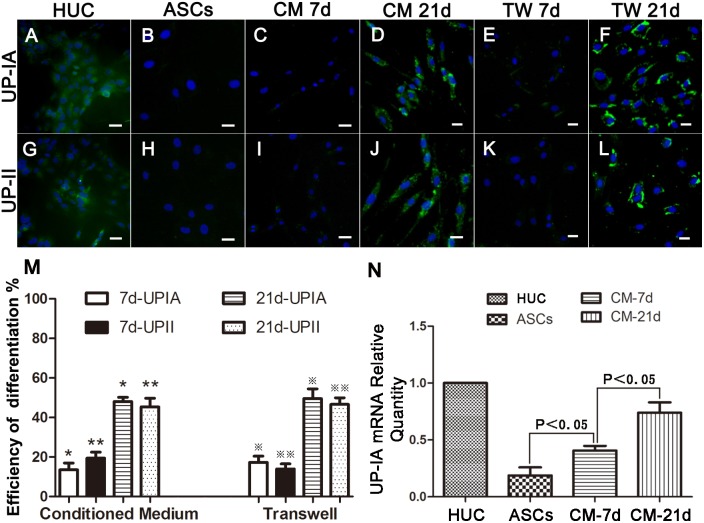
Urothelium-like phenotype differentiation of ASCs. Immunofluorescent staining detection of UP-IA and UP-II in differentiated ASCs. The differentiation efficiency of induction by conditioned medium and transwell system for 7 and 21 days. The quantification of mRNA for the urothelium-specific marker UP-IA in ASCs after induction by conditioned media for 7 and 21 days. A, G: UP-IA and UP-II expression in urothelium. B, H: ASCs did not express UP-IA or UP-II. C, I, E, K: ASCs expressed UP-IA and UP-II in a small amount after induction by CM or transwell system for 7 days. D, J, F, L: UP-IA and UP-II expression in ASCs after induction for 21 days by conditioned medium or transwell system. M: The difference in the ASC differentiation efficiency between the two different induction methods at the 7^th^ and 21^st^ day. N: The quantification of UP-IA mRNA in human ASCs after induction by conditioned media for 7 and 21 days. CM = conditioned medium, TW = transwell indirect co-culture, Scale bar = 20 µm.

HUCs observed using an inverted phase contrast microscope demonstrated a polygonal, cobblestone-shaped morphology (Fig. 1A). Immunofluorescent staining showed that HUCs (Fig. 2 A, G) but not ASCs (Fig. 2 B, H) expressed the urothelium-specific markers UP-IA and UP-II.

The adherent monolayer of ASCs exhibited a spindle-shaped morphology (Fig. 1B) and covered approximately 90% of the culture plate after incubation in a dish for 2 weeks. ASCs induced by conditioned medium (Fig. 1 C) and induced by indirect co-culture transwell system for 21 days (Fig. 1 D) exhibited a different morphology compare with non-induced ASCs. After 21 days induction, the ASCs demonstrated a polygonal morphology and rich in cytoplasm. But they did not show cobblestone-shaped morphology like urothelium. By flow cytometric analysis of ASCs at passage number 3, expression of mesenchymal stem cell markers, including CD29 (99.84%), CD44 (99.77%) and CD105 (97.32%), was confirmed. There was lower expression of CD45 (0.16%) and CD34 (0.26%) (Fig. 1E).

After osteogenic induction for 3 weeks, the ASCs were positively stained with alizarin red for calcium phosphate precipitates (Fig. 1F), indicating that the cells differentiated into osteoblasts. After adipogenic induction for 2 weeks, the ASCs were positively stained for Oil Red O, indicating the formation of intracellular lipid droplets (Fig. 1G). These results suggested that the ASCs have multipotency and could be used for further in vitro differentiation studies.

### Urothelium Specific Marker Expression in ASCs after Induction

The native ASCs did not express UP-IA and UP-II (Fig. 2 B, H) before induction. After 7 days induction by CM, we observed that ASCs expressed UP-IA and UP-II, markers specific for urothelium, in a small amount (Fig. 2 C, I). The expression of UP-IA and UP-II significantly increased in ASCs induced by CM for 21 days compared to those induced for 7 days (Fig. 2 D, J). When indirectly co-cultured with HUCs through a transwell system for 7 and 21 days, ASCs expressed UP-IA and UP-II in the same manner (Fig. 2 E, K, F, L). We evaluated the number of ASCs that expressed UP-IA and UP-II after 7 and 21 days by sampling the two induction manners in vitro. After 7 days induction by CM, 13.54±2.00% of the ASCs expressed UP-IA and 19.49±1.73% expressed UP-II. After induction for 21 days, 48.03±1.25% of the ASCs expressed UP-IA and 45.38±2.50% expressed UP-II. By the transwell induction method, the expression rates of uroplakins in ASCs were as follows: 17.28±1.84% of UP-IA and 13.98±1.47% of UP-II at the 7^th^ day, 49.57±2.85% of UP-IA and 46.58±1.95% of UP-II at the 21^st^ day. The expression rate of UP-IA and UP-II were significantly increased after 21 days induction compared with 7 days induction (P<0.05). We found that the induction efficiency had no significant differences (P>0.05) between the two induction methods in vitro (Fig. 2 M). Human dermal fibroblasts were induced in the same conditions, but did not express urothelium specific markers like ASCs. (Fig.S1).

To confirm the expression levels of mRNA of urothelium specific markers in ASCs, we isolated the total cellular RNA of ASCs after induction by CM for 7 and 21 days and performed quantification RT-PCR for UP-IA. We found that UP-IA mRNA expression could be detected in ASCs after CM induction for 7 and 21 days. The quantity of UP-IA mRNA was dramatically increased after induction for 21 days compared to induction for 7 days. (P<0.05) (Fig. 2N).

### The Expression Level of Cytokines and Soluble Receptors

**Figure 3:**
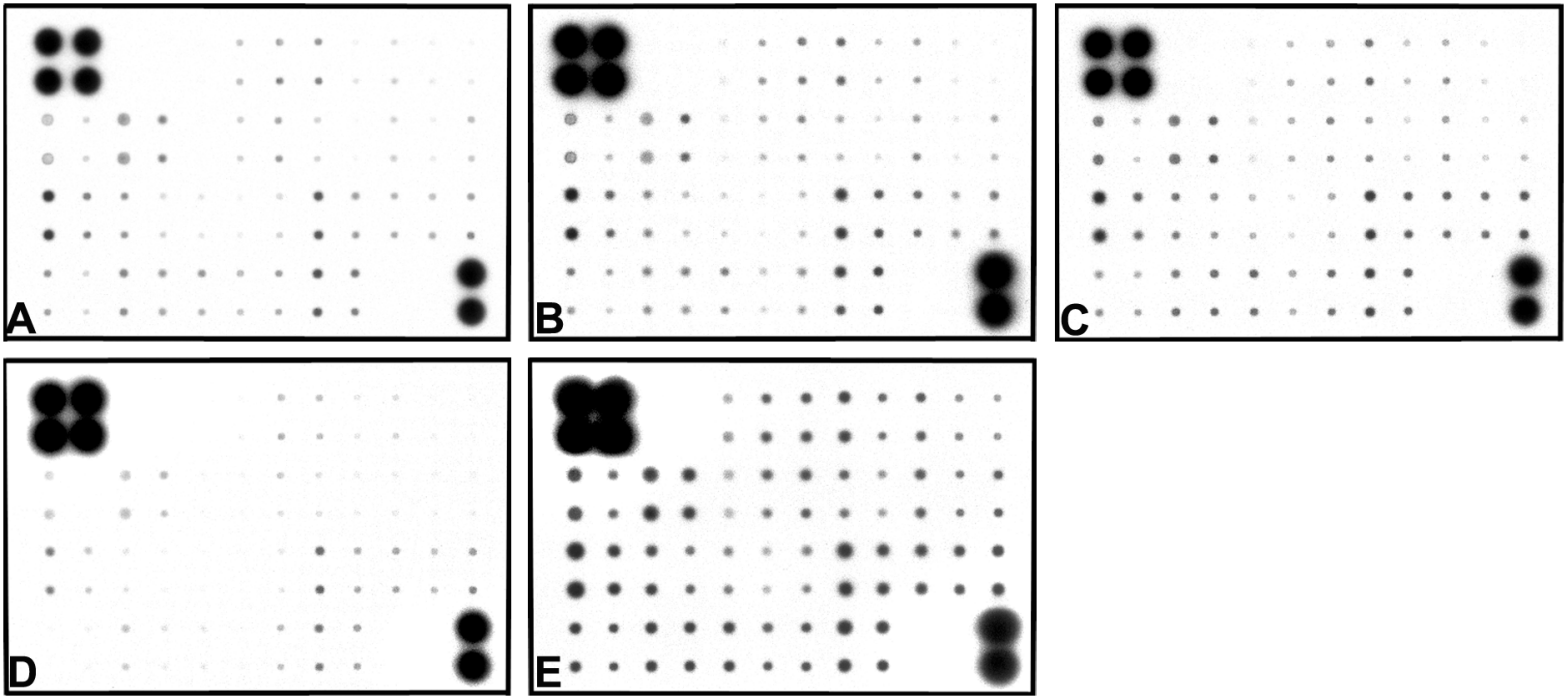
Images of Rabio Human Growth Factor Antibody Array. A total of 41 cytokines were placed on the array. Samples were collected from non-induced ASCs (A) and culture medium from ASCs induced for 12 hours (B), 7 days (C), 21 days (D) and CM from HUC (E).

**Figure 4:**
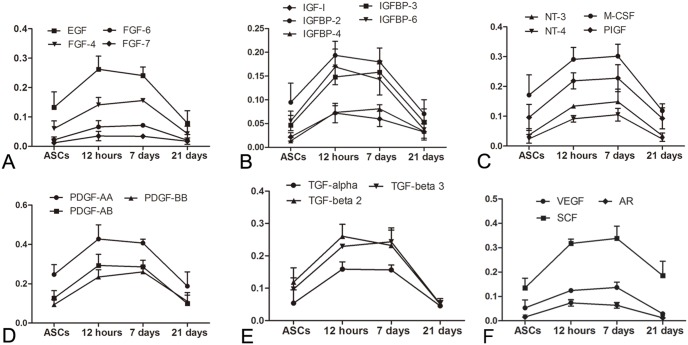
The tendency of dynamic changes of the 22 cytokines. The tendency of dynamic changes of the 22 cytokines at different time points during ASC differentiation towards urothelium-like cells after induction of ASCs for 0 hours, 12 hours, 7 days and 21 days. A: EGF, FGF-4, FGF-6, and FGF-7. B: IGF-I, IGFBP-2, IGFBP-3, IGFBP-4, and IGFBP-6. C: NT-3, NT-4, M-CSF, and PlGF. D: PDGF-AA, PDGF-BB, and PDGF-AB. E: TGF-alpha, TGF-beta 2, and TGF-beta 3. F: AR, VEGF and SCF.

To study the dynamic changes of soluble cytokines and receptors, 41 cytokines and cytokine receptors were detected by the Human Cytokine Antibody Array G Series in the upper medium of urothelium, ASCs induced for 12 hours, 7 days and 21 days and non-induced ASCs. Cytokine dot position on the membrane of Human Growth Factor Antibody Array and the results are shown in Table 1–3 and Fig. 3. We found 36 cytokines and cytokine receptors in the upper medium of urothelium that had significantly higher expression than the non-induced ASCs medium (Table 2), including 28 cytokines and 8 cytokine receptors. After 7 days induction of ASC, 30 cytokines and cytokine receptors were significantly increased in the upper medium compared to the non-induced ASCs medium (P<0.05), including 22 cytokines and 8 cytokine receptors (Table 3). We analyzed the dynamic changes of the 22 cytokines at the different time points during ASC differentiation towards urothelium and plotted the tendency curves of these cytokines (Fig. 4). We found that the relative content of the cytokines in the upper medium of non-induced ASCs was at a low level. All of the cytokines increased sharply at 12 hours and remained at a high level after induction for 7 days; however, the cytokines declined markedly at the 21st day of induction.

**Table 1:** Cytokine position on membrane of Human Growth Factor Antibody Array.

	A	B	C	D	E	F	G	H	I	J	K	L
1	POS	POS	NEG	NEG	AR	bFGF	b-NGF	EGF	EGF R	FGF-4	FGF-6	FGF-7
2	POS	POS	NEG	NEG	AR	bFGF	b-NGF	EGF	EGF R	FGF-4	FGF-6	FGF-7
3	GCSF	GDNF	GM-CSF	HB-EGF	HGF	IGFBP-1	IGFBP-2	IGFBP-3	IGFBP-4	IGFBP-6	IGF-I	IGF-I SR
4	GCSF	GDNF	GM-CSF	HB-EGF	HGF	IGFBP-1	IGFBP-2	IGFBP-3	IGFBP-4	IGFBP-6	IGF-I	IGF-I SR
5	IGF-II	M-CSF	M-CSF	NT-3	NT-4	PDGF Rα	PDGF Rβ	PDGF-AA	PDGF-AB	PDGF-BB	PlGF	SCF
6	IGF-II	M-CSF	M-CSF	NT-3	NT-4	PDGF Rα	PDGF Rβ	PDGF-AA	PDGF-AB	PDGF-BB	PlGF	SCF
7	SCF R	TGF-α	TGF-β	TGF-β2	TGF-β3	VEGF	VEGF R2	VEGF R3	VEGF-D	BLANK	BLANK	POS
8	SCF R	TGF-α	TGF-β	TGF-β2	TGF-β3	VEGF	VEGF R2	VEGF R3	VEGF-D	BLANK	BLANK	POS

**Table 2:** The difference and non-difference cytokines and cytokine receptors that were measured in the upper medium of HUCs compared to non-induced ASCs.

Difference				Non- Difference
FGF basic	HB-EGF	*M-CSF R*	SCF	AR
beta NGF	IGFBP-2	NT-3	*SCF R*	GDNF
EGF	IGFBP-3	NT-4	TGF-beta 1	HGF
*EGF R*	IGFBP-4	*PDGF R alpha*	TGF-beta 2	IGFBP-1
FGF-4	IGFBP-6	*PDGF R beta*	TGF-beta 3	TGF-alpha
FGF-6	IGF-I	PDGF-AA	VEGF	
FGF-7	*IGF-I SR*	PDGF-AB	*VEGF R2*	
GCSF	IGF-II	PDGF-BB	*VEGF R3*	
GM-CSF	M-CSF	PlGF	VEGF-D	

(Italic text: cytokine receptors; Normal text: cytokines).

**Table 3:** The difference and non-difference cytokines and cytokine receptors measured in the upper medium of ASCs induced for 7 days compared to non-induced ASCs.

Difference			Non-Difference	
AR	IGF-I	PDGF-BB	FGF basic	VEGF-D
EGF	*IGF-I SR*	PIGF	Beta NGF	
*EGF R*	M-CSF	SCF	GCSF	
FGF-4	*M-CSF R*	*SCF-R*	GDNF	
FGF-6	NT-3	TGF-alpha	GM-CSF	
FGF-7	NT-4	TGF-beta2	HB-EGF	
IGFBP-2	*PDGF R alpha*	TGF-beta3	HGF	
IGFBP-3	*PDGF R beta*	VEGF	IGPBP-I	
IGFBP-4	PDGF-AA	*VEGF R2*	IGF-II	
IGFBP-6	PDGF-AB	*VEGF R3*	TGF-beta 1	

(Italic text: cytokine receptors; Normal text: cytokines).

**Figure 5:**
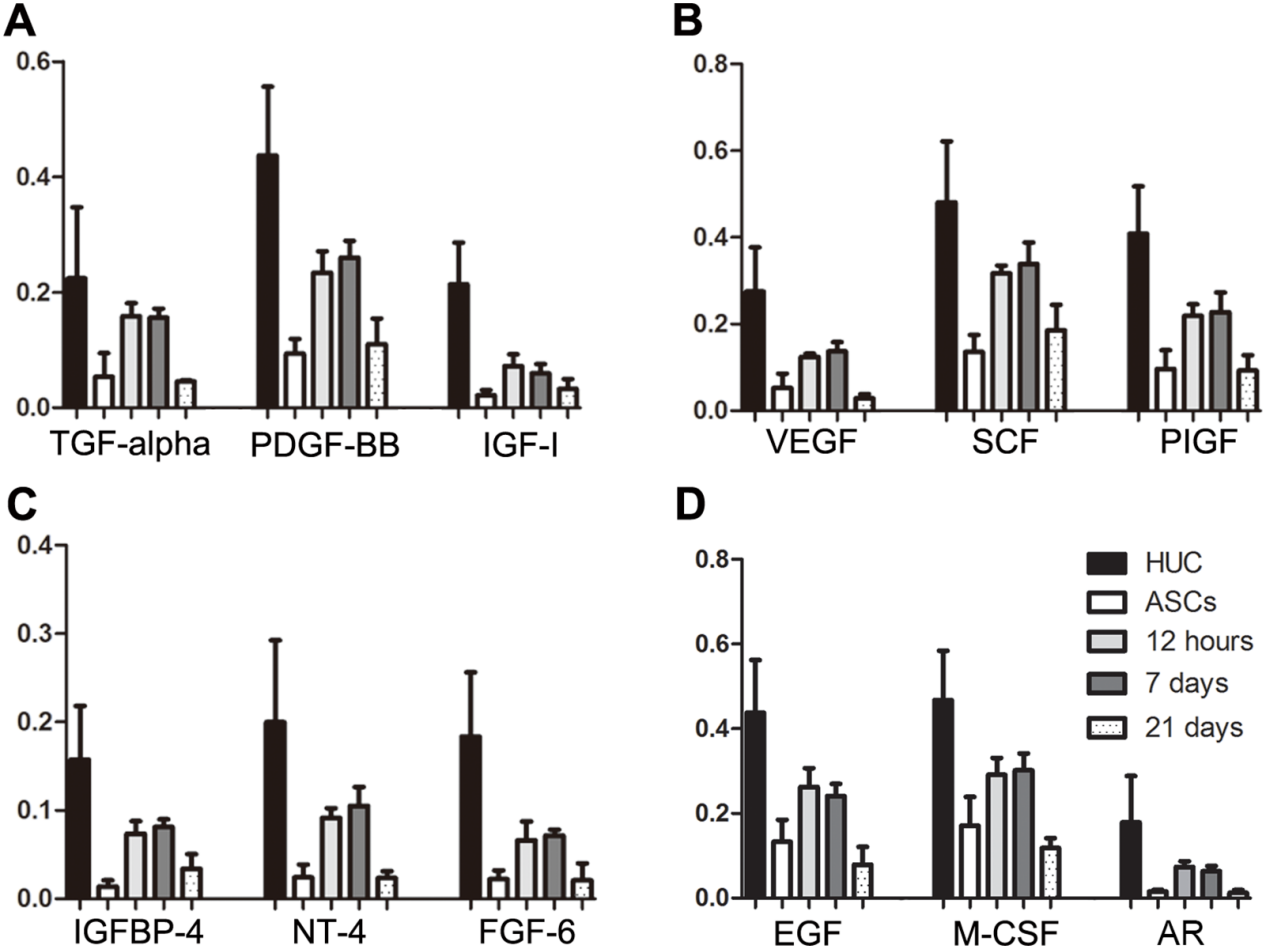
The relative content of the 12 cytokines in the upper medium of HUCs and ASCs differentiated to urothelium at different time points. A: TGF-alpha, PDGF-BB, and IGF-I; B: VEGF, SCF, and PIGF; C: IGFBP-4, NT-4, and FGF-6; D: AR, EGF, M-CSF.

Included in the 22 cytokines were 12 cytokines that had the largest difference of relative content in their respective cytokine classes at the different induction time points. These 12 cytokines are as follows: AR, TGF-alpha, PDGF-BB, IGF-I, VEGF, SCF, PIGF, IGFBP-4, NT-4, FGF-6, EGF and M-CSF. We calculated the relative contents and plotted the trends of these 12 cytokines during the different time points of ASC differentiation to urothelium (Fig. 5). The cytokine levels showed no difference between ASC induction for 12 hours and 7 days (P>0.05). In this study, we also found 8 soluble cytokine receptors, including EGF R, IGF-I SR, M-CSF R, PDGF R alpha, PDGF R beta, SCF-R, VEGF R2 and VEGF R3, that were increased significantly in the upper medium of induced ASCs compared with non-induced ASCs. Six soluble cytokines and cytokine receptor pairs were elevated in the upper media of induced ASCs, including EGF/EGFR, IGF-I/IGF-I SR, M-CSF/M-CSF R, PDGF-AA, PDGF-AB, PDGF-BB/PDGF R alpha, PDGF R beta, SCF/SCFR, VEGF/VEGF R2 and VEGF R3.

## Discussion

ASCs have multi-lineage differentiation ability. They could be induced to adipocytes, osteocytes, chondrocytes, smooth muscle cells, neurocytes etc. [1], [3]. Recently, ASCs had also been used for bladder regeneration [2], [5]. Most of the studies were mainly focused on inducing ASCs toward smooth muscle cells [5], [15], while few studies involved inducing ASCs towards urothelium. Because the urothelium is derived from the endoderm, whereas bone marrow stem cells (BMSCs) or ASCs are derived from the mesoderm, the cross-mesoderm differentiation of urothelium from ASCs is more difficult than ASC differentiation towards smooth muscle cells, which are derived from mesoderm[14], [16], [17]. Our previous in vivo study found that ASCs expressed UP-IA and UP-II partially when co-implanted with urothelium into the subcutaneous tissue of athymic mice after 2 weeks [7], which demonstrated that ASCs have the potential to be induced to urothelium-like cells in vivo.

Co-culturing and using CM from target mature cells are efficient ways to induce adult stem cell towards tissue specific terminal differentiated cells in vitro. Tian et al [14] found that BMSCs could be differentiated towards urothelium by CM and the indirect transwell system. Liu and his colleague [9] have shown that ASCs can be differentiated into a urothelial phenotype using a cell-to-cell contact method but not using indirect co-culture or CM methods. However, Shi et al [8] found that ASCs could be differentiated into a urothelial phenotype by a CM method. In this study, we used the method of both urothelium derived CM and indirect co-culture with urothelium through a transwell system to induce ASC differentiation into urothelium-like cells in vitro for 7 and 21 days. The induction results showed that the ASCs expressed urothelium specific markers UP-IA and UP-II at both the protein and mRNA levels. As a negative control, we used the same inducing conditions to induce human dermal fibroblasts from normal foreskin at passage number 3 as negative control and did not find any expression of uroplakin markers. We also have found the culture medium of F12K supplemented with serum at a concentration of 10% could not induce ASCs differentiation towards urothelium after 4 weeks in our previous study (data not shown). Our study has demonstrated that ASCs can be induced towards a urothelial phenotype using either CM or indirect co-culture methods. Combined with our previous results [7], both in vitro and in vivo results have demonstrated that ASCs can be differentiated towards a urothelial phenotype.

The involved mechanism of ASC differentiation towards urothelium is still under investigation. In our previous study and Liu’s research, cell-to-cell contact between ASCs and urothelium played an important role in the differentiation process [7], [9], [18]. However, in this in vitro system with no cell-to-cell contact, the soluble cytokine growth factors from HUCs in CM appear to be the factors that trigger ASCs to differentiate towards a urothelial phenotype. Paracrine signaling is a key mechanism for the directed differentiation of adult stem cell towards urothelium. Tian and Shi [8], [14] both detected the limiting cytokines of differentiation of BMSCs and ASCs towards urothelium, including EGF, VEGF, PDGF-BB and TGF-β1. They found that PDGF-BB and VEGF expression were significantly higher in the conditioned media of induced BMSCs and ASCs than in the non-induced control. The results suggested the CM from urothelium could alter the microenvironment of both BMSCs and ASCs and initiate their differentiation towards urothelium. However, we believe the complicated cross-mesoderm differentiation process should involve more inducing signals. We detected 41 cytokines and cytokine receptors in the CM and the upper medium of non-induced ASCs. The results demonstrated that the levels of 28 cytokines and 8 cytokine receptors in CM were significantly higher than in the upper medium of non-induced ASCs, indicating that these cytokines and receptors play important roles in initiating the directed differentiation of ASCs towards urothelium. We suppose the above cytokines from CM switched on the urothelium differentiation of ASCs by paracrine. Like the mesendoderm induction of ESCs by exogenous and paracrine signals[19], an integration network of numerous growth factors promote the process of urothelium-like differentiation of ASCs.

In addition to paracrine signaling, autocrine signaling is another common method for the differentiation of mesenchymal stem cells[20], [21]. Because the medium was replaced with fresh serum-free medium 12 h before collection, the differences in the expression of soluble cytokines and receptors among the ASCs induced for 12 h, 7 days and 21 days and the non-induced ASCs can reflect the autocrine signaling of ASCs. The autocrine signaling of ASCs may maintain the differentiation phenotype of ASCs after the trigger of the initial urothelial differentiation process. The differences in the expression of 22 cytokines and 8 receptors between the ASCs induced for 7 days and the non-induced control can show the autocrine signaling of ASCs during differentiation triggered by the 28 cytokines from HUC medium. From the 22 cytokines, we chose 12 cytokines that had the largest differences in expression levels in their respective cytokine classes between induced and non-induced ASCs, including AR, TGF-alpha, PDGF-BB, IGF-I, VEGF, SCF, PIGF, IGFBP-4, NT-4, FGF-6, EGF and M-CSF; they are involved in various signaling pathways during cell growth, proliferation and differentiation. Except for VEGF and PDGF-BB, which have been shown to have key differentiation roles in previous studies [8], [14], there were many other cytokines also involved in ASC differentiation towards urothelium in this study. Additionally, we observed the expression of 8 soluble cytokine receptors and 6 cytokine and cytokine receptor pairs that were elevated in induced ASCs. The screened candidate cytokines and receptors will provide us more clues for further in vitro differentiation studies.

The dynamic change pattern is also critical to enhance the ASC differentiation rate in vitro in the future. All of the cytokines in the culture medium of induced ASCs had a similar dynamic change pattern compared to non-induced ASCs. The cytokines increased at 12 hours, remained at a high level at 7 days and decreased at 21 days. Most of the stem cell differentiation process has temporal changes. For instance, both the autocrine and paracrine signal acted in the early stage of adipogenic differentiation process as early as 12 hours[21]. After the initial and persistent paracrine inducing signals by HUCs, the autocrine signaling of induced ASCs was elevated to a higher level at 12 hours and 7 days and declined significantly at 21 days. The temporal change in the levels of cytokines and receptors indicated that the differentiation process of ASCs towards urothelium is more dependent on cytokine signaling in the early and intermediate stages of 12 hours and 7 days than in the later stage of 3 weeks. The similar finding has been illustrated by Jackson and his colleagues, in which they found once ESCs pass through a temporal window by combined growth factors would no longer dependent on the previous induction pathway[19]. Also the ASCs can be irreversibly differentiated towards an osteogenic lineage after 6 to 9 days[22]–[24].

The relationship between paracrine and autocrine signal regulation during ASCs differentiation to urothelium is complicated. Both the autocrine and paracrine factor are believed as necessary conditions for the differentiation process of ASCs into adipocytes[21]. They used an adipogenic medium (AM) or a conditioned medium (CM) to induce ASCs into adipocytes. The results inferred the AM stimulate the ASCs to secrete an unknown factor acting as a positive regulating signal in the adipogenic differentiation process. Combined with previous literatures and our results, we suppose various cytokines in CM switch on the urothelium-like cell differentiation process by a paracrine signal. Then the ASCs secrete similar cytokines to maintain urothelium differentiation by autocrine. The temporal change of cytokines indicated that the differentiation process of ASCs towards urothelium is more dependent on cytokine signaling in the early and intermediate stages of 12 hours and 7 days than the later stage of 3 weeks.

Combining different cytokines to induce certain stem cells toward a committed lineage is a common differentiation strategy in vitro [12], [25]. Our next study will detect the key signal pathways in the urothelium inducing of ASCs and use different cytokines and soluble receptors in combination to effectively induce ASCs towards urothelium. Furthermore, we will demonstrate the roles of cytokines and receptors in directed differentiation by regulating their expression in vitro. Both a combination stimulation using various paracrine soluble cytokines and stable autocrine signaling by ASCs in the early and intermediate stage of differentiation are promising urothelium inducing strategies.

## Conclusion

ASCs have a potential to be differentiated into urothelium-like cells in vitro using both CM and a transwell co-culture system with mature urothelial cells. Numerous cytokines and soluble receptors are involved in the differentiation process with dynamic temporal changes. This result indicates that the induction of ASCs towards urothelium-like cells is a sophisticated procedure involving both paracrine and autocrine signaling. Further studies should be carried out to determine the detailed regulation mechanism of cytokines and receptors and to enhance the urothelium differentiation efficiency of ASCs.

## Supporting Information

Figure S1 **Immunofluorescent staining detection of UP-IA and UP-II in human dermal fibroblasts induced for 21 days by CM and transwell system.** A: UP-IA did not express on fibroblasts after induced for 21 days by CM. B: UP-IA did not express on fibroblasts after induced for 21 days by transwell. C: UP-II did not express on fibroblasts after induced for 21 days by CM. D: UP-II did not express on fibroblasts after induced for 21 days by transwell. TW = transwell indirect co-culture. Scale bar = 20 µm.
(TIF)

Table S1 **Abbreviation of the 41 cytokines and the cytokine receptors detected by Human Cytokine Antibody Array G Series.**
(DOCX)
