# Supplementary figures and images for: The Differentiation of Human Adipose-Derived Stem Cells towards a Urothelium-Like Phenotype In Vitro and the Dynamic Temporal Changes of Related Cytokines by Both Paracrine and Autocrine Signal Regulation
Source: PLoS One. 2014 Apr 21;9(4):e95583. doi: 10.1371/journal.pone.0095583 (PMC3994076; doi:10.1371/journal.pone.0095583)

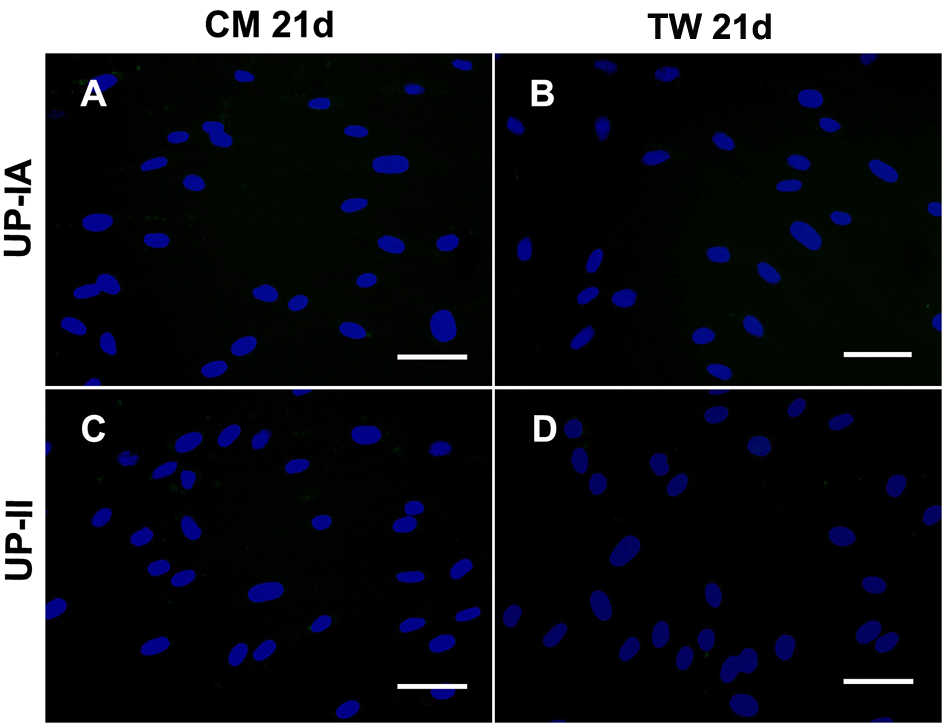

Supplement: Figure S1 — Immunofluorescent staining detection of UP-IA and UP-II in human dermal fibroblasts induced for 21 days by CM and transwell system. A: UP-IA did not express on fibroblasts after induced for 21 days by CM. B: UP-IA did not express on fibroblasts after induced for 21 days by transwell. C: UP-II did not express on fibroblasts after induced for 21 days by CM. D: UP-II did not express on fibroblasts after induced for 21 days by transwell. TW = transwell indirect co-culture. Scale bar = 20 µm. (TIF) [file pone.0095583.s001.tif]
