# Supplementary material for: The Differentiation of Human Adipose-Derived Stem Cells towards a Urothelium-Like Phenotype In Vitro and the Dynamic Temporal Changes of Related Cytokines by Both Paracrine and Autocrine Signal Regulation
Source: PLoS One. 2014 Apr 21;9(4):e95583. doi: 10.1371/journal.pone.0095583 (PMC3994076; doi:10.1371/journal.pone.0095583)
Supplement: Table S1 — Abbreviation of the 41 cytokines and the cytokine receptors detected by Human Cytokine Antibody Array G Series. (DOCX) [file pone.0095583.s002.docx]

**Table S1 Abbreviation of the 41 cytokines and the cytokine receptors detected by Human Cytokine Antibody Array G Series**

| Abbreviation of cytokines and the cytokines receptors | |
| --- | --- |
| AR= amphiregulin | M-CSF=macrophage colony-stimulating factor |
| FGF basic=fibroblast growth factor basic | M-CSF R=macrophage colony-stimulating factor receptor |
| beta NGF=beta nerve growth factor | NT-3=neurotrophin-3 |
| EGF=epidermal growth factor | NT-4=neurotrophin-4 |
| EGF R=epidermal growth factor receptor | PDGF R alpha=platelet-derived growth factor receptor alpha |
| FGF-4=fibroblast growth factor 4 | PDGF R beta=platelet-derived growth factor receptor beta |
| FGF-6=fibroblast growth factor 6 | PDGF-AA=platelet-derived growth factor AA |
| FGF-7=fibroblast growth factor 7 | PDGF-AB=platelet-derived growth factor AB |
| GCSF=granulocyte colony-stimulating factor | PDGF-BB=platelet-derived growth factor BB |
| GDNF=glial-cell-line derived neurotrophic factor | PlGF=placental growth factor |
| GM-CSF=granulocyte-macrophage colony-stimulating factor | SCF=stem cell factor |
| HB-EGF=heparin-binding EGF-like growth factor | SCF R=stem cell factor receptor |
| HGF=hepatocyte growth factor | TGF-alpha=transforming growth factor-alpha |
| IGFBP-1=insulin-like growth factor binding protein-1 | TGF-beta 1=transforming growth factor-beta 1 |
| IGFBP-2=insulin-like growth factor binding protein-2 | TGF-beta 2=transforming growth factor-beta 2 |
| IGFBP-3=insulin-like growth factor binding protein-3 | TGF-beta 3=transforming growth factor-beta 3 |
| IGFBP-4=insulin-like growth factor binding protein-4 | VEGF=vascular endothelial growth factor |
| IGFBP-6=insulin-like growth factor binding protein-6 | VEGF R2=vascular endothelial growth factor receptor 2 |
| IGF-I=insulin-like growth factor-I | VEGF R3=vascular endothelial growth factor receptor 3 |
| IGF-I SR=insulin-like growth factor-I soluble receptor | VEGF-D=vascular endothelial growth factor D |
| IGF-II=insulin-like growth factor-II |  |
